# Subcutaneous in situ gel delivered leuprolide acetate's consistent and prolonged drug delivery maintains effective testosterone suppression independent of age and weight in men with prostate cancer

**DOI:** 10.1002/bco2.13

**Published:** 2020-04-22

**Authors:** Joseph F. Renzulli, Scott T. Tagawa, Stuart N. Atkinson, Deborah M. Boldt‐Houle, Judd W. Moul

**Affiliations:** ^1^ Urology Yale School of Medicine New London CT USA; ^2^ Medical Oncology and Urological Oncology Weill Cornell Medicine New York NY USA; ^3^ Medical Affairs Tolmar Pharmaceuticals, Inc Buffalo Grove IL USA; ^4^ Urology Duke University School of Medicine Durham NC USA

**Keywords:** androgen deprivation therapy, castration‐resistance prostate cancer, leuprolide acetate, pharmacokinetics, prostate cancer

## Abstract

**Objectives:**

To assess the impact of patient age and weight on the pharmacokinetics (PK), testosterone (T) suppression and safety from four fixed dosing regimens (7.5, 22.5, 30, or 45 mg for 1‐, 3‐, 4‐, or 6‐months, respectively) of subcutaneous in situ gel delivered leuprolide acetate (Gel‐LA) injected via the ATRIGEL Delivery System in patients with prostate cancer (PCa).

**Patients and methods:**

Two patient populations were specified for analysis: a small cohort of surgically castrated PCa patients and a large, pooled population of PCa patients from four pivotal trials of Gel‐LA. Two separate analyses of the impact of age and weight on study endpoints were conducted: (1) PK and safety of a single monthly dose of Gel‐LA in a Phase 1 study with PCa patients who had undergone bilateral surgical orchiectomy (“Bilaterally orchiectomized male study”); (2) PK/pharmacodynamic (PD) effects and safety using pooled data from four pivotal trials assessing 1‐, 3‐, 4‐, and 6‐month dosing of Gel‐LA in patients with advanced PCa, stratified by age and body weight (pivotal trials).

**Results:**

Eight orchiectomized patients from the “Bilaterally orchiectomized male study” and 438 patients from the pivotal trials were included in the analyses. Age and body weight did not appear to affect the PK results in the orchiectomized patient population. Pooled pivotal trial data showed that serum T levels did not appear to be influenced by age or weight; ≥90% of patients across all age groups and ≥92% of patients across all weight groups achieved T ≤ 50 ng/dL by week 4. Median T levels for castration (T ≤ 50 ng/dL) were maintained from week 3 until the end of the study and all subgroups achieved median T ≤ 20 ng/dL by week 4. Patients from the orchiectomized patient study did not report any serious treatment‐related adverse events (AEs) and there were no AE‐related withdrawals from the study. The most common AEs were hot flashes and injection site events. The safety profiles from pivotal trials have been previously described and, as expected, were consistent with known effects of LHRH agonist therapy and suppression of T levels.

**Conclusion:**

PK and PD of Gel‐LA appear to be unaffected by age and body weight, as demonstrated by persistence of effective drug levels through the dosing period and consistent T suppression across different ages and body weights.

## 1 INTRODUCTION

Prostate cancer (PCa) is the second most common cancer in men, with 1.3 million affected globally in 2018, and an anticipated 165 000 cases with 29 000 deaths per year in the United States.^1^, ^2^ PCa is an androgen‐dependent tumor that proliferates in the presence of testosterone (T),^3^ and androgen deprivation therapy (ADT) is the gold standard for initial treatment of advanced PCa. A highly effective and inexpensive treatment used worldwide for rapidly reducing T levels is surgical castration by bilateral orchiectomy. However, in countries where chemical castration is available, this method is preferred, as surgery is irreversible and has potential for psychological distress.^4^ Luteinizing hormone‐releasing hormone (LHRH) agonists such as leuprolide acetate (LA), triptorelin, and goserelin are the most commonly used therapies to suppress T to castrate level.^5^ Degarelix, an LHRH antagonist, is an alternative agent for T suppression through a different mechanism. The target T level for castration has historically been defined as <50 ng/dL. However, modern assay methodologies have determined T levels after surgical castration are closer to 15 ng/dL.^6^ In 2014 the European Association of Urology guidelines defined the target for T suppression during ADT as ≤20 ng/dL.^7^ Draft guidance in 2019 from the U.S. Food and Drug Administration (FDA) includes T < 20 ng/dL as a secondary efficacy endpoint in trials of new gonadotropin‐releasing hormone analogues and requests those data be included in labels.^8^ Achievement of this lower T level has been shown to correlate with delayed onset of castration‐resistant PCa and improved clinical outcomes such as disease‐specific survival.^3^, ^9^, ^10^, ^11^, ^12^

Upon initiation of ADT with an LHRH agonist, elevation of luteinizing hormone (LH) and follicle‐stimulating hormones lead to a significant increase (surge) in T that may result in a clinical flare. Though the surge typically decreases within a few weeks of dosing, it can be hazardous to some patients by worsening symptoms such as bone pain, neuropathy, hematuria, and urethral or bladder outlet obstruction due to stimulation of cancer cells.^13^ These agents have also been associated with “end‐of‐dose phenomena,” that is, premature exhaustion of effective drug release resulting in acute‐on‐chronic effects with a rise in T to above castrate levels following a subsequent dose, which may lead to disease progression.^6^ To reduce these risks, it is important to select a treatment with an extended duration of action to deliver effective T suppression levels that are maintained until the patient receives a subsequent dose. Maintenance of effective drug levels beyond the dosing interval would be of particular value when the timing of a subsequent dose is delayed due to circumstances such as patient illness, inclement weather, office scheduling complications, vacations, etc. As it is important to maintain castration T levels during ADT, it is also of interest to understand if factors such as age or body weight should be considered when selecting a therapy.

Leuprolide acetate is a synthetic nonapeptide LHRH agonist indicated for the palliative treatment of advanced PCa^13^ that induces downregulation of pituitary GnRH receptors, reduction in release of LH and suppression of steroidogenesis in the testes when delivered continuously.^14^ One formulation of LA, which is available in 1‐, 3‐, 4‐, or 6‐month doses, is an in situ gel (LA for injectable suspension, Gel‐LA) administered subcutaneously via the ATRIGEL Delivery System, a biodegradable poly(lactide‐co‐glycolide) (PLGA) system that enables consistent‐controlled release of the active ingredient.^13^, ^15^ T production is suppressed after the initial surge following the first injection of Gel‐LA and T remains below castrate levels with each subsequent injection. In pivotal trials, clinical efficacy of the four doses of Gel‐LA demonstrated continuous suppression of T to concentrations ≤20 ng/dL through achievement of median serum levels of LA above 0.05 ng/mL (assay lower limit of quantification [LLOQ]: 0.1 ng/mL for 1‐ and 3‐month and 0.05 ng/mL for 4‐ and 6‐month formulations).^16^, ^17^, ^18^, ^19^, ^20^ The pharmacokinetics (PK) of a single dose of Gel‐LA have also previously been described in healthy subjects (“PKPD study”).^21^

Luteinizing hormone‐releasing hormone agonists are administered as fixed doses with formulations differing by duration of dosing. However, it has not been established whether patient‐specific factors such as age or body weight may affect serum levels of drug or resultant efficacy. Data from a PK and safety study of single dose Gel‐LA in patients with advanced PCa who had undergone bilateral orchiectomy are presented. These PK data provide support for the results reported in pivotal trials, and the safety profile of LA in these patients should represent effects of the drug itself, as events related to T suppression will likely be a consequence of their previous surgery. A previously published report of four pivotal trials of the 1‐, 3‐, 4‐, and 6‐month doses of Gel‐LA examined the PK and pharmacodynamics (PD) of Gel‐LA.^16^, ^17^, ^18^, ^19^, ^20^ In the present analysis, we evaluated PK of Gel‐LA and potential impact of age and body weight on T suppression via two analyses: (1) PK of a single monthly dose of Gel‐LA in PCa patients who had undergone bilateral surgical orchiectomy (“Bilaterally orchiectomized male study”) and (2) PK/pharmacodynamic (PD) effects from four pivotal trials in patients with advanced PCa, stratified by age and body weight (pivotal trials). PK results from the “PKPD study” are also mentioned for the purpose of comparison.

## 2 MATERIALS AND METHODS

All study protocols, amendments, and informed consent were reviewed and approved by the study centers’ institutional review board (IRB). All studies were conducted according to Good Clinical Practice guidelines, IRB regulations, and informed consent regulations, in accordance with the Declaration of Helsinki.

### 2.1 Phase 1 trial of Gel‐LA in bilaterally orchiectomized men

An 8‐week, open‐label, single‐arm study was performed with the objective of evaluating safety, tolerability, and PK following a single dose of 7.5 mg Gel‐LA in surgically castrated men with advanced PCa. Testosterone levels were not evaluated for this study population.

Men aged 45‐85 years with histologically or cytologically proven adenocarcinoma of the prostate who had undergone bilateral orchiectomy at least 2 months prior to study start were eligible. Patients had to provide written informed consent, be within 25% of the ideal weight for their age, have a World Health Organization (WHO)/Eastern Cooperative Oncology Group performance status of 0‐2, serum T ≤ 50 ng/dL at screening, and have a life expectancy of at least 9 months. Exclusion criteria included hospitalization, serious concurrent illness or disease, evidence of brain metastases, spinal cord compression, or urinary tract obstruction. Patients were also excluded if they had received immunotherapy, radiotherapy, chemotherapy, or surgery (except transurethral resection of the prostate or radical prostatectomy) within the prior 2 months, hormonal therapy within 2 months of baseline, or were anticipated to receive concomitant hormonal therapy for PCa during the 2 months after baseline.

All patients received a single dose of 7.5 mg LA suspended in the ATRIGEL Delivery System (34% w/w Poly [DL‐lactide‐co‐glycolide] and 66% w/w N‐methyl‐2‐pyrrolidone) as a subcutaneous injection into the upper right or upper left quadrant of the abdomen. Blood samples for Gel‐LA PK analysis were collected at baseline, at 15 and 30 minutes, at 1, 2, 3, 4, 6, 8, 10, 12, 24, 36, 48, and 72 hours, and on days 4, 7, 14, 21, 28, 35, 42, 49, and 56 post injection. LA concentrations were determined with solid‐phase extraction and high‐performance liquid chromatography followed by radioimmunoassay. LLOQ of LA was 0.1 ng/mL for this study. Safety was evaluated by adverse event (AE) reporting and clinical laboratory measurements (hematology, coagulation, serum chemistry, and urinalysis) at screening, 24 and 72 hours, and days 7, 14, 28, and 56. Vital signs (body weight, heart rate, blood pressure, respiratory rate, and temperature) were recorded at screening, baseline, 0.5, 4, 12, 24, 48, and 72 hours, and days 28 and 56. Follow‐up duration was 56 days.

#### 2.1.1 Statistical analysis

Serum concentrations of LA were summarized as mean, standard deviation (SD), % relative SD, median, minimum, and maximum levels. PK parameters included the maximum observed LA concentration (C_max_), time of maximum serum concentration (T_max_), time of last measured LA concentration in serum (tldc), and area under the LA serum concentration vs time curve (AUC). AUC was determined by linear trapezoidal interpolation, and actual times of blood draws were used for the PK analyses. Summary statistics for PK parameters were performed for all patients combined. Clinical laboratory tests and AEs were analyzed using descriptive statistics (mean and SD) where possible. Due to the small sample size of this study, analyses based on weight and age were not performed as they would be insufficiently powered to derive any strong conclusions.

### 2.2 Pivotal trials of Gel‐LA in advanced PCa

Data collected from four prospective, open‐label, fixed‐dose clinical trials of Gel‐LA in patients with advanced PCa were pooled as previously described.^16^, ^17^, ^18^, ^19^, ^20^ Patients were aged 40‐86 years and had not previously received ADT. Patients were required to have a histological or cytological diagnosis of prostate adenocarcinoma (stage >T1), WHO performance score 0‐2 and life expectancy ≥1 year. Patients were excluded at baseline if they had serum T < 150 ng/dL, PCa therapy in the preceding 2 months, prostatic surgery in the preceding 2 weeks, or hormone therapy in the preceding 3 months. Other eligibility criteria have been previously described.^16^, ^17^, ^18^, ^19^, ^20^ Patients received one of four Gel‐LA formulations: (1) 7.5 mg LA once every 28 days for 24 weeks (1‐month formulation; n = 120); (2) 22.5 mg LA once every 84 days for 24 weeks (3‐month formulation; n = 117); (3) 30 mg LA once every 112 days for 32 weeks (4‐month formulation; n = 90); (4) 45 mg LA once every 24 weeks for 48 weeks (6‐month formulation; n = 111).

Serum T levels were determined using radioimmunoassay. Serum LA levels were determined using radioimmunoassay for the 1‐ and 3‐month formulation study and mass spectrometry for the 4‐ and 6‐month formulation studies. T measurements were taken 2‐4 times on day 0 and once on days 1, 2, 3, 7, and every 7 days thereafter until the next scheduled dose. The patients receiving 45 mg had an additional measurement taken on day 2.

#### 2.2.1 Statistical analysis

Serum LA concentrations were determined for PK analysis (eg, C_max_, T_max_, and AUC). Descriptive statistics (eg, mean, standard error, median, minimum, and maximum) were used to summarize the LA concentrations at each time point and T suppression. Patients were stratified by age subgroups (age <60 years, 60‐<70 years, 70‐<80 years, and ≥80 years) and body weight subgroups (<70 kg, 70‐<80 kg, 80‐<90 kg, 90‐<100 kg, 100‐<110 kg, 110‐<120 kg, and >120 kg) for analyses.

### 2.3 Safety

For all pivotal trials of Gel‐LA and the “Bilaterally orchiectomized male study,” safety was measured through the collection of adverse event (AE) information at all visits beginning with the baseline visit.

## 3 RESULTS

### 3.1 Baseline characteristics in “Bilaterally orchiectomized male study” and pivotal studies

All patients had advanced PCa (Jewett stage C1, C2, D1, or D2), and baseline characteristics and demographics were similar for both populations (Table 1). The Phase 1 trial (“Bilaterally orchiectomized male study”) enrolled eight men with a mean age of 73 years (range 61‐79 years) and mean weight of 88 kg (range 69‐105 kg) (Table 1). Across the four pivotal trials, a total of 438 eugonadal PCa patients, ages 46‐86 years and weight 49‐146 kg were enrolled. The mean ages and weights of patients were comparable across trials (mean age range across trials: 73‐74 years; mean weight range across trials: 84‐89 kg; Table 1). Caucasian patients accounted for 100% of patients in the Phase 1 trial and 76%‐80% of patients in the pivotal trials. Across the four pivotal trials, mean baseline serum T concentration ranged from 361 to 386 ng/dL.

**Table 1:** Patient demographics and baseline characteristics

Parameter	Pivotal trials: dosing regimen				Orchiectomized patient study
	1‐Month	3‐Month	4‐Month	6‐Month	Single dose
	7.5 mg	22.5 mg	30 mg	45 mg	7.5 mg
	(n = 120)	(n = 117)	(n = 90)	(n = 111)	(n = 8)
Age, years, mean (range)	72.8 (52‐85)	73.1 (46‐85)	73.5 (53‐84)	73.2 (50‐86)	72.5 (61‐79)
Age, years, % (n)					
40‐49	0	0.9 (1)	0	0	0
50‐59	6.7 (8)	5.1 (6)	6.7 (6)	5.4 (6)	0
60‐69	23.3 (28)	23.1 (27)	22.2 (20)	22.5 (25)	50.0 (4)
70‐79	50.0 (60)	44.4 (52)	46.7 (42)	49.6 (55)	50.0 (4)
80‐89	20.0 (24)	26.5 (31)	24.4 (22)	22.5 (25)	0
Race, n (%)					
White	76.7 (92)	79.5 (93)	78.9 (71)	75.7 (84)	100 (8)
Black	12.5 (15)	11.1 (13)	11.1 (10)	17.1 (19)	0
Hispanic	10.8 (13)	6.0 (7)	8.9 (8)	5.4 (6)	0
Asian	0	2.6 (3)	0	0.9 (1)	0
Other	0	0.9 (1)	1.1 (1)	0.9 (1)	0
Height, inches, mean (range)	69.0 (62‐75)	68.2 (55‐74)	69.0 (60‐78)	68.9 (62‐76)	70.25 (67‐72)
Weight, kg, mean (range)	84.1(57‐130)	84.4 (59‐134)	89.1 (60‐142)	86.2 (49‐146)	87.7 (69‐105)

Abbreviation: SD, standard deviation.

### 3.2 PK in “Bilaterally orchiectomized male study”

**Figure 1:**
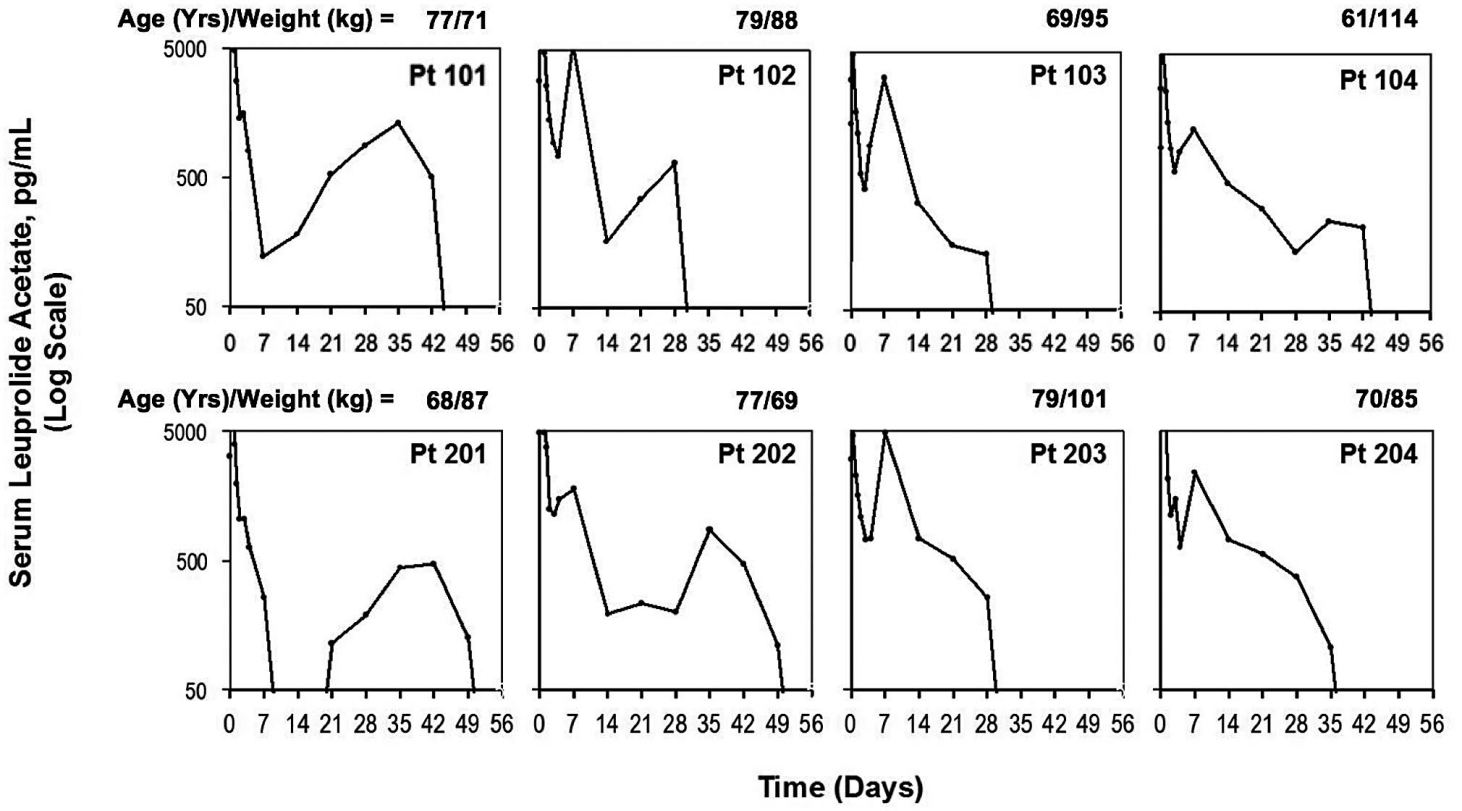
Serum LA concentration over time for individual bilaterally orchiectomized patients, log scale. Blood samples were collected and serum LA concentrations were determined at 15 and 30 minutes, 1, 2, 3, 4, 6, 8, 10, 12, 24, 36, 48, and 72 hours, and days 4, 7, 14, 21, 28, 35, 42, 49, and 56 after injection. The lower limit of quantification (LLOQ) was 0.1 ng/mL. On day 14, LA concentration for Pt 201 below the LLOQ. Pt, patient

**Figure 2:**
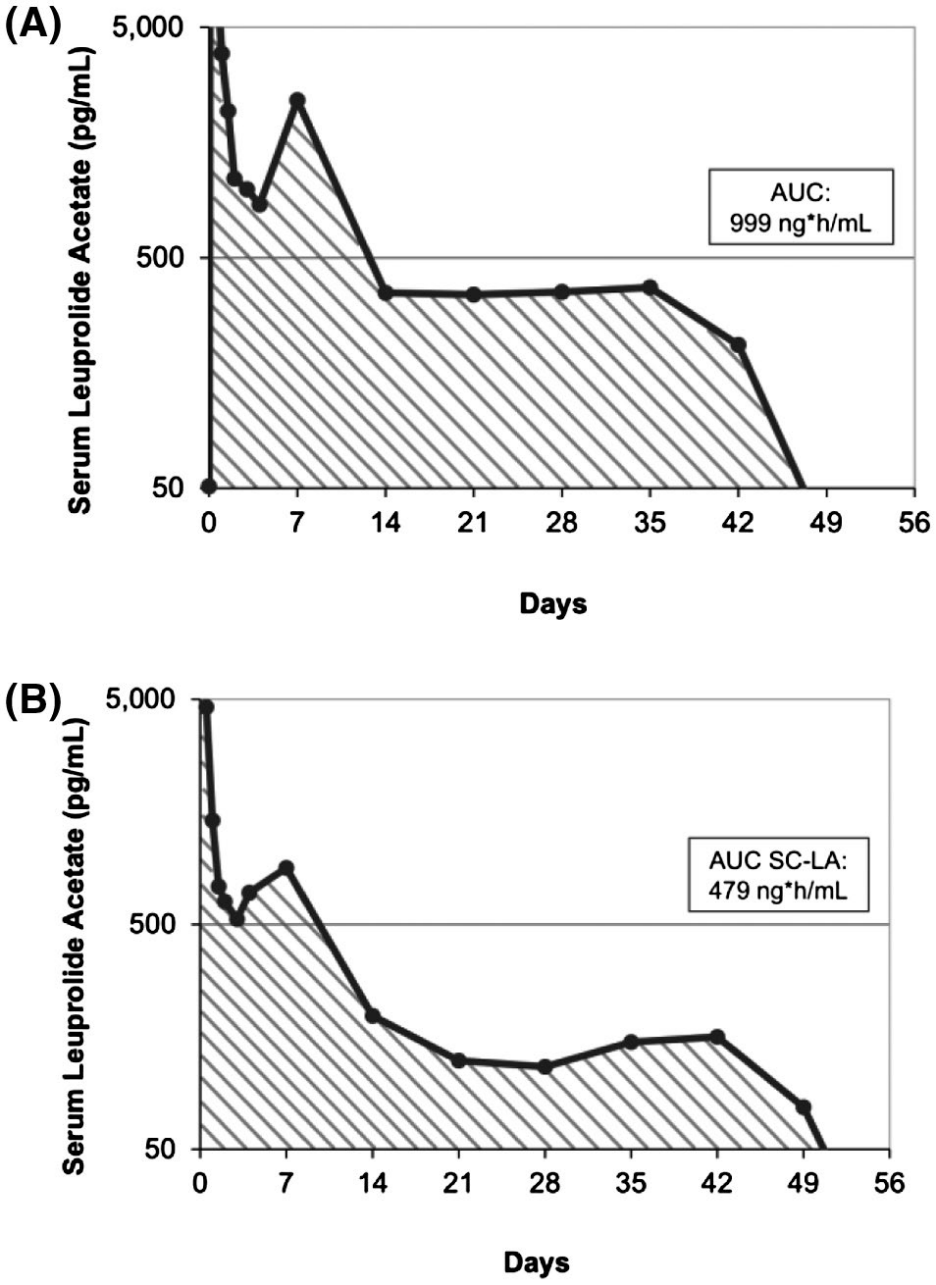
Mean serum LA concentration over time in bilaterally orchiectomized patients compared with healthy subjects, log scale. A, Serum LA over time in bilaterally orchiectomized patients (n = 8). Blood samples were collected and serum LA concentrations were determined at 15 and 30 minutes, 1, 2, 3, 4, 6, 8, 10, 12, 24, 36, 48, and 72 hours, and on Days 4, 7, 14, 21, 28, 35, 42, 49, and 56 after subcutaneous injection. B, Mean serum LA in healthy subjects following a single 7.5 mg injection of Gel‐LA (n = 16) from a Phase 1, open‐label, parallel‐group study. [Copyright permission to be obtained]

Figure 1 shows the serum LA levels over time for each patient in the “Bilaterally orchiectomized male study.” Following an injection of Gel‐LA, there was an initial rapid absorption phase with maximal concentrations of LA observed at 2‐6 hours. Serum LA then decreased slowly over days 2‐4. There was a slight increase in mean LA concentration from days 4‐7 for six of the eight patients, followed by a plateau period during which LA levels slowly declined over 2 weeks, which is common for LHRH agonists. Serum LA was above the LLOQ (0.1 ng/mL) for all patients (n = 8) for a mean of 37 days, a minimum of 28 days and a maximum of 49 days (Figure 2A). LA was above the LLOQ for both patient subgroups with weight ≥193.4 lb (n = 4) and <193.4 lb (n = 4). All patients had detectable LA levels regardless of age (age range 61‐79 years). C_max_ was 26.3 ± 12.6 ng/mL and mean T_max_ was 3.79 ± 1.39 hours. The AUC_0‐tldc_ was 999 ± 247 ng h/mL with low inter‐patient variability (CV = 24%) (Table 2).

**Table 2:** Comparative Pharmacokinetics of LA: Summary of Studies of Orchiectomized Patients, Eugonadal PCa Patients and Healthy Subjects Receiving 7.5 mg (1‐month) Subcutaneous Injections of Gel‐LA

Study population	C_max_, ng/mL	T_max_, hours	AUC_last_, ng h/mL	No. days >LLOQ	Ref.
	Mean (SD)			Mean (range)	
PCa patients					
Orchiectomized (n = 8)	26.3 (12.6)	3.8 (1.4)	999 (247)	37 (28‐49)	
Eugonadal (n = 20)	26.3 (12.6)	4.7 (1.4)	966 (230)	37 (28‐49)	17
Healthy subjects (n = 16)	19 (8.0)	2.1 (0.8)	479 (132.6)	NP (42‐56)	20

Note

Study populations were not directly compared, therefore no statistical analyses were conducted.

Abbreviations: AUC, area under the curve; C_max_, maximal observed concentration; LLOQ, lower limit of quantification; NP, not provided; SD, standard deviation; tldc, time of last determinable concentration; T_max_, time of maximal concentration.

### 3.3 PK in pivotal trials and “PKPD study”

**Figure 3:**
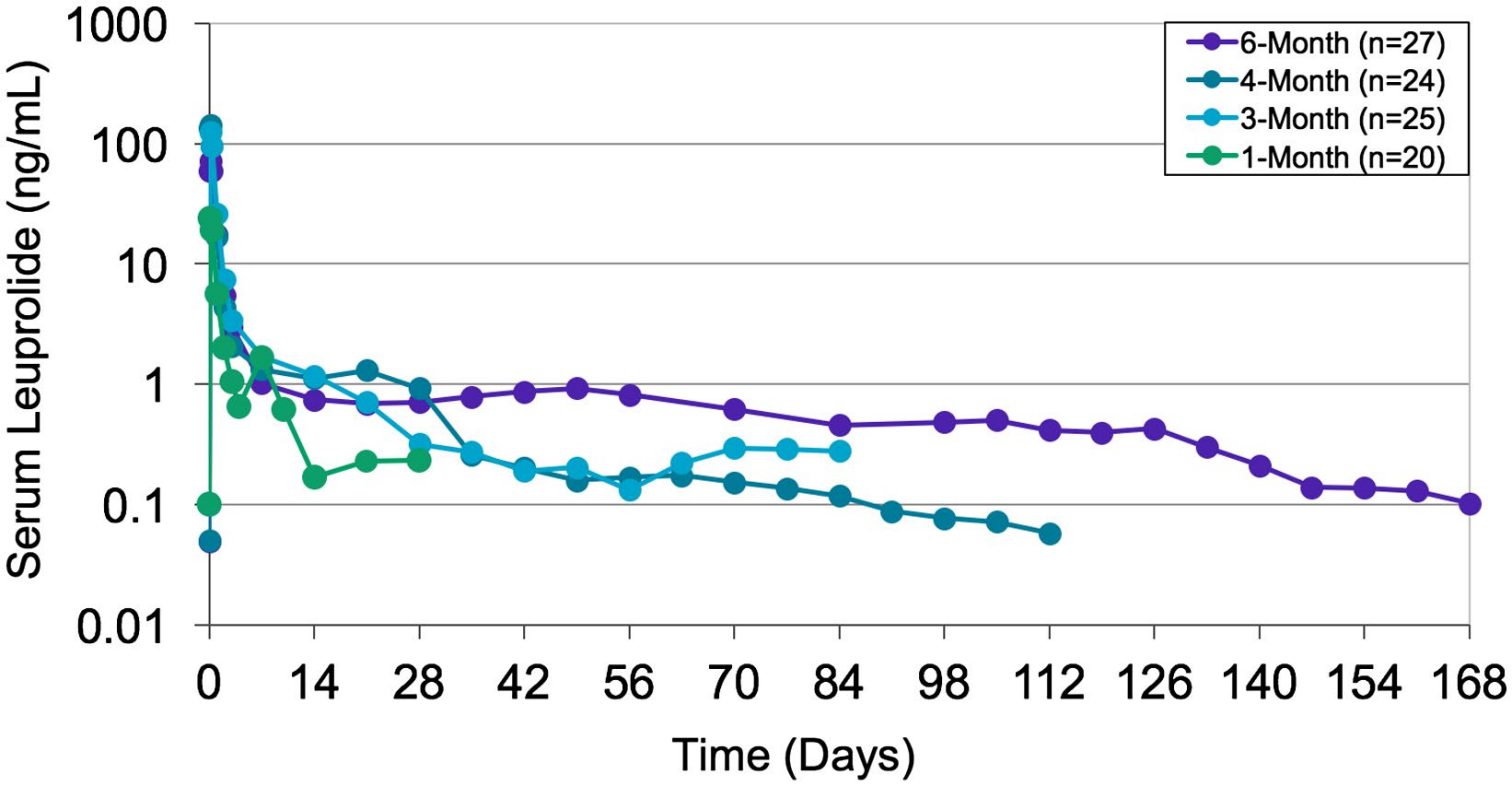
Median serum LA concentration over time in pooled pivotal trials of patients with leuprolide concentrations measured. Serum LA and T levels were determined using liquid chromatography mass spectrometry and radioimmunoassay, respectively. Descriptive statistics summarized the concentration of LA and T at each time point

Figure 3 shows serum LA concentrations over time for patients in the 1‐, 3‐, 4‐, and 6‐month pivotal trials. For all trials, median serum LA levels across all study patients were consistently between 0.05 and 1 ng/mL from week 2 until the end of the studies. The pivotal trial of eugonadal PCa patients (n = 20, PK population) receiving 7.5 mg injections of Gel‐LA over 1 month showed mean C_max_ 26.3 ± 12.6 ng/mL, T_max_ 4.66 ± 1.44 hours and AUC_last_ 966 ± 230 ng h/mL.^1^ Serum LA for all 1‐month eugonadal PCa patients was above the LLOQ of 0.1 ng/mL for at least 28 days with a mean of 37 days (range: 28‐49 days).

A previously published study (“PKPD study”) with a healthy patient population reported C_max_ 19 ± 8.0 ng/mL, T_max_ 2.1 ± 0.8 hours and AUC_last_ 479 ± 132.6 ng h/mL (n = 16) (Table 2). LLOQ (0.05 ng/mL) was exceeded for at least 42 days in the healthy patient population (range: 42‐56 days; mean not reported) (Table 2).

### 3.4 PD by age and weight in pivotal trials

**Figure 4:**
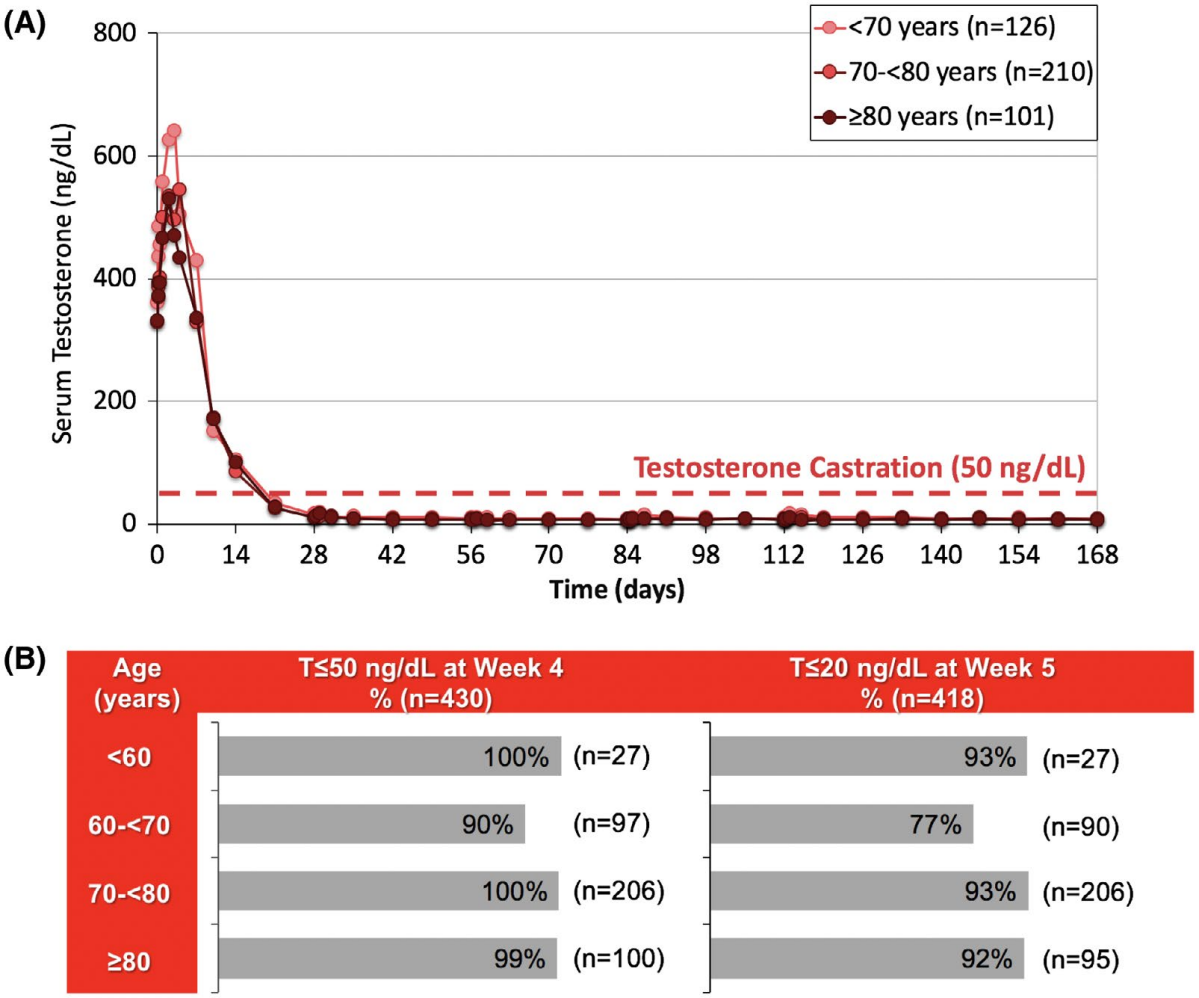
Serum T by patient age in pooled pivotal trials. A, Median serum T over time (n = 437). Error bars represent interquartile ranges; B, Proportion of patients achieving T ≤ 50 ng/mL by Week 4 and T ≤ 20 ng/mL by Week 5 (n = 430)

**Figure 5:**
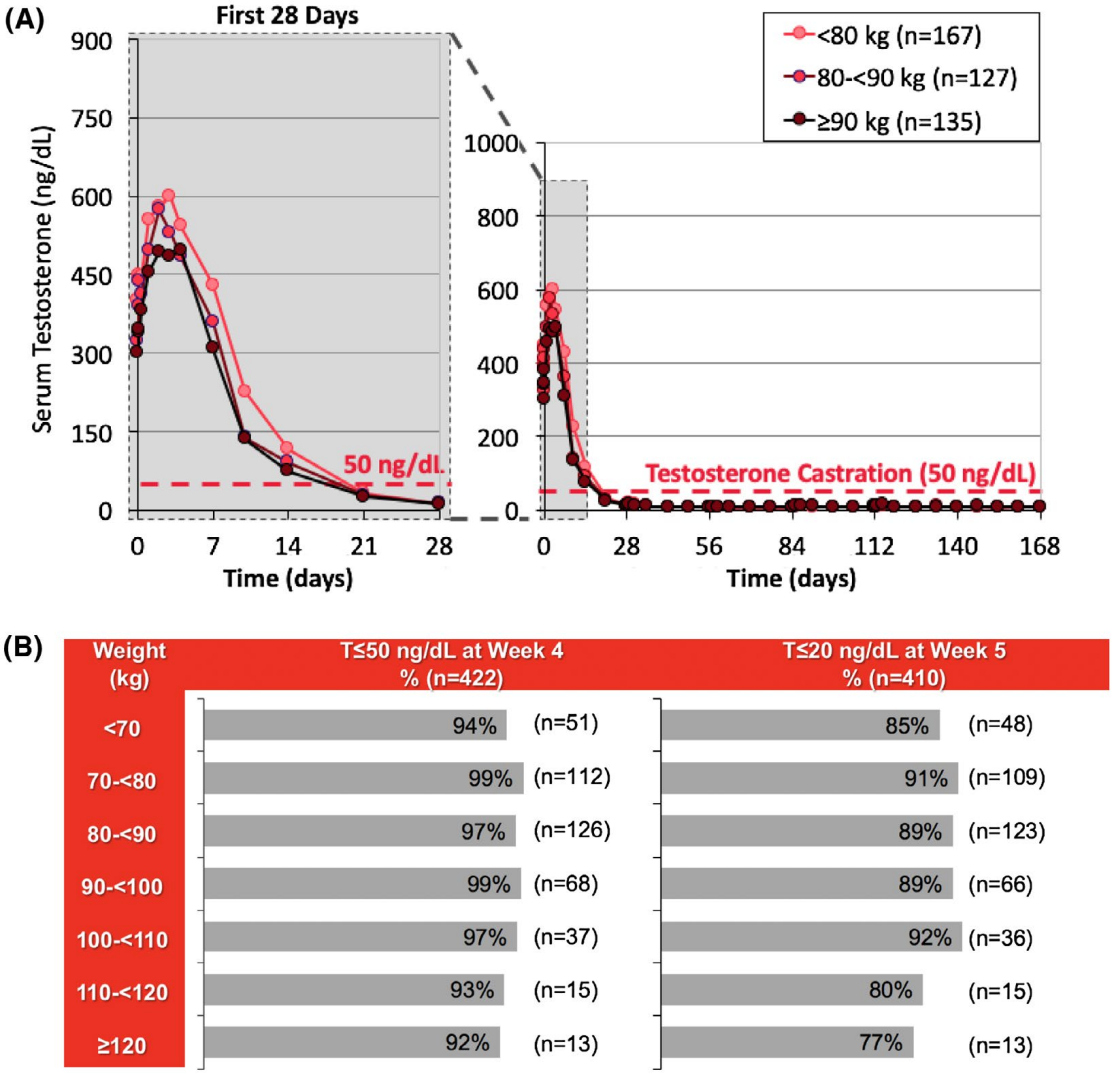
Serum T by patient body weight in pooled pivotal trials. A, Median serum T over time (n = 437); Error bars represent interquartile ranges; B, Proportion of patients achieving T ≤ 50 ng/mL by Week 4 and T ≤ 20 ng/mL >y Week 5

Figures 4 and 5 display serum T levels with patients stratified by age and weight. Median serum T levels for all subgroups remained below castrate levels from week 3 until the end of the study (week 3:34, 26, and 27 ng/dL for age <70, 70‐<80, and ≥80 years, respectively; end of study: 12, 9, and 6 ng/dL for the three age subgroups, respectively) and achieved ≤20 ng/dL by week 4 (Figures 4A and 5A). 100%, 90%, 100%, and 99% of patients aged <60 years, 60‐70 years, 70‐80 years, and ≥80 years achieved T ≤ 50 ng/dL by week 4, respectively (Figure 4B). About 94% to 99% of patients in weight subgroups <110 kg, 93% of patients weighing 110‐120 kg, and 92% of patients ≥120 kg achieved T ≤ 50 ng/dL by week 4 (Figure 5B).

### 3.5 Safety in “Bilaterally orchiectomized male study” and pivotal trials

In the “Bilaterally orchiectomized male study,” 31 AEs were reported, the most common of which was discomfort upon injection (88%; n = 7), described as mild and transient, which lasted a few seconds in six patients and for less than a few minutes in one patient. Other AEs occurring in >1 patient included mild bruising at the injection site (38%; n = 3), mild hot flashes (25%; n = 2), and two (25%) cases of severe gastrointestinal disturbances, both of which were deemed unrelated to study treatment. No serious treatment‐related AEs were reported, and no patients discontinued the study due to AE. No clinically relevant laboratory abnormalities or changes in vital signs were noted over the course of the study.

The safety profiles in the pivotal trials of Gel‐LA have been previously described and, as expected, were consistent with known effects of LHRH agonist therapy.^20^

## 4 DISCUSSION

This evaluation studied the impact of age and weight on the PK of single dose Gel‐LA in orchiectomized PCa patients and on T suppression in a pooled analysis from four pivotal trials in patients with advanced disease. Neither age nor body weight appeared to affect PK of LA or T suppression in the majority of patients following Gel‐LA dosing. This interpretation is supported by findings suggesting persistence of effective LA levels through the dosing period and consistent T suppression across different ages and body weights.

### 4.1 PK in “Bilaterally orchiectomized male study”

Injection of a single 7.5 mg dose of Gel‐LA in orchiectomized patients with adenocarcinoma of the prostate delivered constant release of effective levels of active drug and a safety and tolerability profile that supports once‐monthly dosing. The profile of serum LA concentration over time during the first 2 weeks after injection was similar between patients, with most exhibiting a small increase around day 10, followed by steady and consistent drug release. Serum LA levels were above the LLOQ throughout, and often beyond, the 1‐month dosing interval up to 49 days for all patients across all ages and body weights, suggesting PK and consequent efficacy may not be adversely affected by these patient factors. Small differences in PK between individuals in the present “Bilaterally orchiectomized male study” may be attributable to differences in body composition or body chemistry of each patient.

Studying bilaterally orchiectomized men with PCa allows understanding of attributes of Gel‐LA, such as the PK including the time point when serum LA becomes undetectable without compromising sustained T suppression to castrate levels. Extended delivery of LA not only confirms clinical benefit in reduced risk of T escape, but also has practical implications, such as providing confidence of continued suppression through the dosing period, in case of delays in timing of a subsequent dose. Additionally, maintenance of these levels provides confidence that acute‐on‐chronic elevations of T are less likely to occur if a subsequent injection is significantly delayed or in cases when insurers mandate dosing based on a calendar month schedule rather than intervals of weeks, as used in the clinical trials. This is particularly relevant for the 6‐month formulation as the differential between 6 calendar months and 24 weeks is the largest (2 weeks) compared to other formulations. Hence, with selection of 6‐month Gel‐LA, both clinicians and patients could benefit from requiring only two office visits per year with increased confidence of continued effectiveness.

### 4.2 PK comparisons across studies (“Bilaterally orchiectomized male study,” pivotal trials, and “PKPD study”)

Although direct statistical comparisons of PK parameters were not conducted as the populations were different in each trial, the overall PK profiles from each study (“Bilaterally orchiectomized male study,” pivotal trials, and “PKPD study”) were comparable. The observation for serum LA level in bilaterally orchiectomized males is consistent with the Gel‐LA clinical study in healthy subjects (“PKPD study”), where duration of measurable and adequate serum LA levels similarly ranged from 42‐56 days after dosing (Table 2, Figure 2B), exceeding the 1‐month dosing period.^21^

In the pivotal trials, consistent and prolonged delivery of leuprolide above 0.05 ng/mL was achieved across all doses. The consistent T suppression across ages and weight subgroups in pivotal trials suggests drug delivery is not affected by these patient‐specific variables. PK findings in PCa patients were similar to those observed in healthy subjects, though a 2‐fold increase in AUC was seen in the PCa populations. Possible reasons for lower AUC in healthy volunteers include faster absorption and metabolism. Overall, results support continued and reliable drug release throughout the labelled dosing intervals. The sustained release of LA due to the ATRIGEL Delivery System might contribute to the consistent drug concentrations regardless of the population's characteristics.^22^

### 4.3 PD in pivotal trials

In the pivotal trials of Gel‐LA for advanced PCa, consistent and prolonged drug delivery above 0.05 ng/mL was achieved across all doses with from week 6 through week 24, while favorable efficacy (T suppression below 20 ng/dL) was also observed over this period.^16^, ^17^, ^18^, ^19^, ^20^ T suppression ≤20 ng/dL was achieved and maintained across all age and weight subgroups, including those with the lowest age (<60 years) and highest weight (>120 kg) (Figures 4 and 5). This was consistent with a previously published pooled analysis across four formulations, in which 90%‐96% of patients (n = 438) achieved T ≤ 20 ng/dL by week 6, and 90%‐97% maintained T ≤ 20 ng/dL from weeks 6 to 24.^17^ It has also been reported that patients with body mass index greater than 35 kg/m^2^ have elevated T levels, suggesting that this population may be at risk for T escape.^16^ Our findings provide evidence that Gel‐LA provides high levels of efficacy throughout dosing intervals regardless of patient age and/or weight.

### 4.4 Safety

The most common treatment‐related AE’s in the “Bilaterally orchiectomized male study,” other than injection discomfort and bruising, were mild hot flashes (n = 2). The low rate is not surprising in surgically castrated patients, where causes may be delayed side effects from orchiectomy, fluctuations in residual T suppression (eg, from adrenal secretion), estradiol deficiency, or stress from study procedures rather than any effect of LA.^23^ The full safety profiles of Gel‐LA in the pivotal trials have been described in the previously published manuscripts.^16^, ^17^, ^18^, ^19^, ^20^

### 4.5 Limitations

One limitation was the small sample size in the bilaterally orchiectomized patient study. Also, the nature of the study population enrolled eliminated potential efficacy endpoints such as reduction in T levels, and no patients with a BMI more than one standard deviation away from normal were entered. The open‐label design and retrospective nature of the pooled pivotal trial analyses were additional limitations. Although this study only analyzed the PK of the 1‐month formulation, all four formulations are composed of the ATRIGEL Delivery System, which is designed to provide controlled and sustained drug release. Therefore, it is likely that the 3‐, 4‐, and 6‐month formulations would also have extended drug delivery beyond the dosing periods as seen with the 1‐month formulation.^24^, ^25^, ^26^ As T suppression was consistently low across all age and/or weight subgroups by the end of the study, and it would be unlikely for the T levels to increase immediately after the last study visit, PK is likely to be consistent across all age and/or weight subgroups until or even beyond the end of the dosing periods. Additionally, results from the pivotal trials may not be generalizable to morbidly obese patients due to the small number of patients ≥110 kg enrolled into the studies.

## 5 CONCLUSIONS

The data presented in this paper demonstrated reliable and persistent drug delivery with a single dose of 7.5 mg Gel‐LA, extending throughout and often beyond the 1‐month dosing period across different ages and body weights. PD results in the pivotal trials proved that the T suppression was achieved and maintained, regardless of age and weight, across all dosing formulations. Taken together, the totality of the data analyzed from the studies suggest that Gel‐LA has a consistent PK profile and achieves very effective T suppression to ≤20 ng/dL without obvious effects due to patient weight and age, providing evidence that individualization of dosing is likely unnecessary.

## DISCLOSURES

JFR has served in a consulting or advisory role for Astellas, Bayer, Ferring, GenomeDx, Janssen, Sanofi, and Tolmar. STT has served in a consulting or advisory role for AbbVie, Astellas, Bayer, Dendreon, Endocyte, Genentech, Immunomedics, Janssen, Karyopharm Therapeutics, Medivation, Sanofi, Seattle Genetics, Amgen, Clovis Oncology, and Tolmar. SNA and DMB are employees of Tolmar Pharmaceuticals. JWM has served in a consulting or advisory role for AbbVie, Astellas, Bayer, Dendreon, Ferring, GenomeDx, Genomic Health, Janssen, Medivation, and Tolmar.
